# Neurodevelopmental Phenotypes and Brain Anomalies in Individuals With Heterozygous *SEMA6A* Variants

**DOI:** 10.1111/cge.70197

**Published:** 2026-06-23

**Authors:** Evan Burchfiel, Xiaonan Zhao, Nichole M. Owen, Tia Gordon, Mahshid S. Azamian, Eric C. Kao, Fan Xia, Xi Luo, Jill A. Rosenfeld, Seema R. Lalani, Allison P. Ortega, Steven B. Bleyl, Florence Petit, Sulekha Rajagopolan, Bénédicte Demeer, Meredith K. Gillespie, Lijia Huang, Matthew Osmond, Kym M. Boycott, Kyra E. Stuurman, Marjon A. van Slegtenhorst, Haley Soller, Céline Jost, Aurore Garde, Hana Safraou, Laurence Faivre, Victor Faundes, Daryl A. Scott

**Affiliations:** ^1^ Department of Molecular and Human Genetics Baylor College of Medicine Houston Texas USA; ^2^ Baylor Genetics Houston Texas USA; ^3^ Department of Cell and Gene Therapy Baylor College of Medicine Houston Texas USA; ^4^ Department of Pediatrics, Division of Medical Genetics University of Utah Salt Lake City Utah USA; ^5^ Clinical Genetics Department Univ. Lille, CHU Lille Lille France; ^6^ Department of Clinical Genetics Liverpool Hospital Liverpool New South Wales Australia; ^7^ Centre Hospitalier Universitaire de Amiens‐Picardie Amiens France; ^8^ Chimere INSERM UA21 University of Picardie‐Jules Verne Amiens France; ^9^ Department of Genetics Children's Hospital of Eastern Ontario Ottawa Ontario Canada; ^10^ Children's Hospital of Eastern Ontario Research Institute University of Ottawa Ottawa Ontario Canada; ^11^ Department of Clinical Genetics Erasmus MC University Medical Center Rotterdam the Netherlands; ^12^ Division of Genetics and Genomic Medicine UPMC Children's Hospital of Pittsburgh Pittsburgh Pennsylvania USA; ^13^ Université Bourgogne Europe, CHU Dijon Bourgogne, Inserm, CTM UMR1231 Dijon France; ^14^ Laboratoire de Génomique Médicale, Inserm, UMR1231, équipe GAD Université Bourgogne Europe, CHU Dijon Bourgogne Dijon France; ^15^ Laboratorio de Genética y Enfermedades Metabólicas, Instituto de Nutrición y Tecnología de los Alimentos Universidad de Chile Santiago Chile

**Keywords:** attention deficit disorder, autism spectrum disorder, CNS anomalies, developmental delay, neurodevelopmental disorder, *SEMA6A*

## Abstract

SEMA6A is a transmembrane protein that plays a role in axon guidance and cell migration. *Sema6a* null mice have cerebral anatomical defects and altered social interactions and working memory. However, the phenotypes associated with loss of SEMA6A function have not been clearly defined in humans. Here we describe 11 individuals who are heterozygous for putatively damaging variants affecting *SEMA6A*. All of these individuals (100%) had neurodevelopmental phenotypes that included developmental delay, intellectual disability, and/or autism spectrum disorder. Abnormal behaviors were seen in 73% with oppositional defiant disorder being diagnosed in 27% and acting out, overeating, and tantrums each being described in 18% of individuals. Disorders of attention were documented in 45%. Among the six individuals who had a brain MRI, 50% had at least one abnormal finding. Of the eight *SEMA6A* variants with known inheritance, five were inherited. Taken together, our data suggest that loss of SEMA6A function may be associated with an increased risk of neurodevelopmental phenotypes, abnormal behaviors, disorders of attention, and brain anomalies. Additional studies will be needed to determine if *SEMA6A* haploinsufficiency is best characterized as an autosomal dominant disorder with incomplete penetrance or as a risk factor for these phenotypes.

## Graphical abstract

SEMA6A plays a role in cell migration and axon guidance in the developing central nervous system. Phenotypes seen in eleven individuals heterozygous for *SEMA6A* variants included developmental delay, intellectual disability, autism/autistic behaviors, behavioral abnormalities, attention disorders, hypotonia, and brain anomalies. Some individuals with *SEMA6A* haploinsufficiency are mildly symptomatic or asymptomatic.

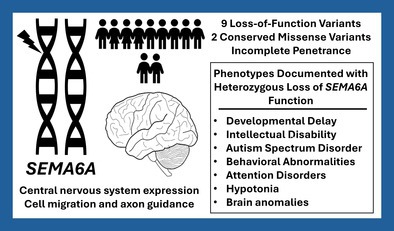

## 1 Introduction

Semaphorin proteins act as guidance signals in embryogenesis and organogenesis [1]. *SEMA6A* codes for a transmembrane semaphorin protein that functions as a homodimer [2, 3]. SEMA6A is highly expressed in the developing neural tissue and is required for normal development of the thalamocortical projection and influences proper hippocampal development in mice [4, 5]. Loss of SEMA6A function in mice causes cerebral anatomical defects, altered social interactions and working memory, loss of GABAergic interneurons, and abnormal oligodendrocyte differentiation [4, 6, 7, 8].

*SEMA6A* has a probability of haploinsufficiency (pHaplo) score of 0.98, a probability of loss‐of‐function intolerance (pLI) score of 1, and a loss‐of‐function observed/expected upper bound fraction (LOEUF) score of 0.36 (gnomAD v4.1.1) [9, 10, 11]. These scores suggest that *SEMA6A* is likely to be a haploinsufficient gene. However, individuals with loss‐of‐function variants in *SEMA6A* are seen in the gnomAD database indicating that some individuals with *SEMA6A* haploinsufficiency are likely to be asymptomatic or mildly symptomatic [9].

Consistent with mouse models, neurodevelopmental phenotypes have been previously described in two individuals with *SEMA6A* loss‐of‐function variants. De Rubeis et al. identified an individual (AC05‐0059‐01) with autism spectrum disorder who was heterozygous for a de novo c.27T>A, p.(Tyr9*) stop‐gain variant in *SEMA6A* [12]. The Deciphering Developmental Disorders (DDD) study included a female (DDD4K.03722) who was heterozygous for a de novo c.2263_2264insC, p.(Leu755Profs*74) frameshift variant in *SEMA6A* [13]. However, this individual was also heterozygous for a de novo pathogenic c.1540_1544dupGGCTT, p.(Phe515Leufs*5) [NM_001319944.2] variant in *BCL11A*, consistent with a diagnosis of Dias‐Logan syndrome (MIM# 617101) which could be contributing to their phenotype.

Here we provide additional evidence that loss of SEMA6A function may be associated with neurodevelopmental phenotypes, abnormal behaviors, disorders of attention, hypotonia, and brain anomalies.

## 2 Materials and Methods

### 2.1 Human Subjects Research

Individuals with variants affecting *SEMA6A* (Subjects S1–S11) were identified using information available in the Baylor Genetics Clinical Database and the DECIPHER database, and through personal communications facilitated by GeneMatcher [14, 15]. Baylor Genetics is a CLIA‐approved, CAP‐certified clinical genetics laboratory. Its clinical database contains data from approximately 20 000 individuals who have undergone exome sequencing and over 100 000 individuals who have had chromosomal microarray analyses. DECIPHER is a publicly accessible database that contains information from over 50 000 individuals submitted from more than 300 centers [14].

Individual research consent was obtained using protocols approved by resident institutional review boards, including Baylor College of Medicine (BCM; H‐18414) and Clinical Trials Ontario (CTO‐1577), or coded/anonymized data are being presented as authorized by the institutional review board of BCM (H‐47546). All research was conducted in accordance with the ethical standards of BCM's committee on human research and international standards.

### 2.2 Variant Annotation

The predicted deleterious potential of *SEMA6A* variants was evaluated using GeneBe (https://genebe.net/) to determine gnomAD allele frequencies and MutationTaster, CADD, and Alpha Missense predictions and scores [16, 17, 18].

Sequence variants were classified by a board‐certified clinical laboratory geneticist based on the 2015 American College of Medical Genetics and Genomics (ACMG) standards for the interpretation of sequence variants (March 2026). The variant of uncertain significance (VUS) category was further subdivided into three sub‐tiers: favoring pathogenic (VUSP), neutral (VUSN), and favoring benign (VUSB) (https://www.baylorgenetics.com/variant‐classification/) [19].

Copy number variants (CNVs) were classified by a board‐certified clinical laboratory geneticist based on the 2020 ACMG and the Clinical Genome Resource (ClinGen) technical standards for the interpretation and reporting of constitutional copy‐number variants [20].

All *SEMA6A* variants are described using NM_020796.5 as a reference. Genomic coordinates are reported based on GRCh38 (hg38) with NC_000005.10 as a reference.

### 2.3 Protein Modeling

The predicted SEMA6A structure was downloaded from the UniProt database (entry Q9H2E6_V1_4; Levy laboratory) [21]. Protein modeling was performed using the pdb file and UCSF ChimeraX v1.11 software [22, 23].

## 3 Results

**FIGURE 1:**
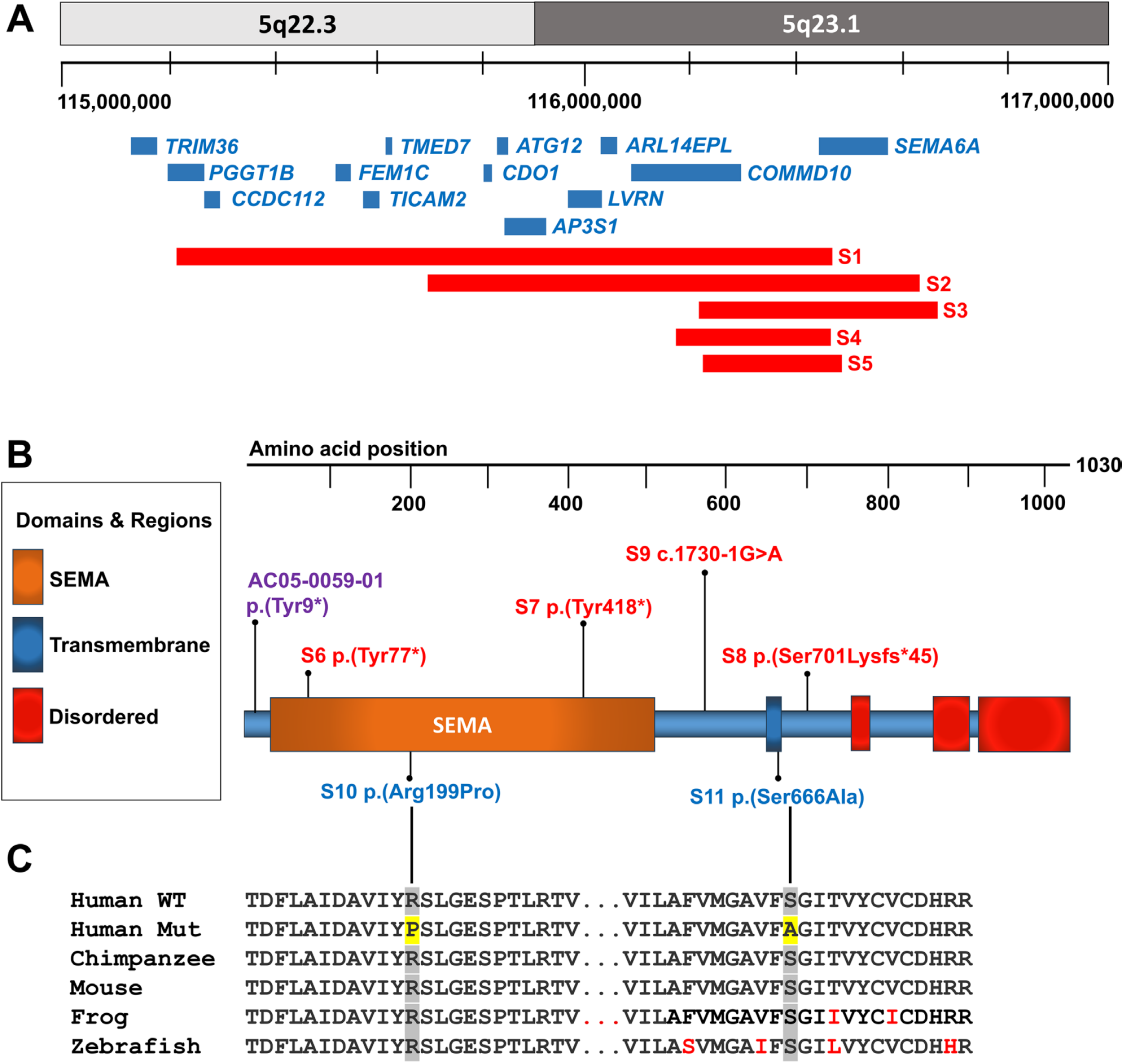
*SEMA6A* variants. (A) The locations of deletions found in Subjects S1–S5 (hg38). (B) The locations of *SEMA6A* sequence variants are shown based on their putative protein location. The stop‐gain variant seen in individual AC05‐0059‐01 reported by De Rubeis et al. is shown in purple [12]. The loss‐of‐function variants seen in Subjects S6–S9 are shown in red, and the missense variants seen in Subjects S10 and S11 are shown in blue. (C) The missense variants (yellow highlight) seen in Subjects S10 and S11 affect conserved residues (gray highlight). Surrounding residues that are not conserved are shown in red. SEMA = semaphorin domain.

We identified five individuals with small deletions (≤ 1.25 Mb) involving *SEMA6A* (Subjects S1‐S5), four individuals with loss‐of‐function variants—two stop‐gain variants (Subjects S6 and S7), a frameshift variant (Subject S8), and one splice variant (Subject S9)—and two individuals with conserved missense variants (Subjects S10 and S11). Their clinical and molecular data are described below and are summarized Table S1 and Figure 1.

Recurrent phenotypes seen in our cohort included neurodevelopmental phenotypes—developmental delay, intellectual disability, and/or autism spectrum disorder (100%, 11/11)—abnormal behaviors (73%, 8/11), disorders of attention (45%, 5/11), and hypotonia (36%, 4/11) (Table 1). Among the six individuals who had a brain MRI, 50% (3/6) had abnormal findings, with dysplastic corpus callosum being the only recurrent finding (33%, 2/6).

**TABLE 1:** Characteristic phenotypic categories in individuals with *SEMA6A* variants.

Subject ID (DECIPHER #)	S1 (275458)	S2 (372234)	S3 (411553)	S4^a^ (294056)	S5	S6	S7^b^	S8^c^	S9	S10	S11		
Age/Sez	3y M	9y F	7y F	13y F	21yM	5y M	17y M	14y M	16y M	4.5y M	11y M		
Sequence change [NM_020796.5]	1.25 Mb Deletion	942 kb Deletion	452 kb Deletion	296 kb Deletion	262 kb Deletion	c.231T>G, p.(Y77*)	c.1254C>G, p.(Y418*)	c.2098_2101dup, p.(S701Kfs*45)	c.1730‐1G>A, p.(?)	c.596G>C, p.(R199P)	c.1996T>G, p.(S666A)		
Inheritance	Non‐maternal	?	?	Maternal	Maternal	De novo	Maternal	Paternal	Maternal	De novo	De novo		
Allele frequency in gnomAD	N/A	N/A	N/A	N/A	N/A	Absent	Absent	Absent	Absent	Absent	Absent		
MutationTaster	N/A	N/A	N/A	N/A	N/A	Disease causing	Disease causing	Disease causing	Disease causing	Disease causing	Disease causing		
CADD	N/A	N/A	N/A	N/A	N/A	41	38	N/A	32	33	25		

												LOF variants; number affected, %	All variants; number affected, %
Alpha missense	N/A	N/A	N/A	N/A	N/A	N/A	N/A	N/A	N/A	1	0.16		
ACMG (VUS subdivision)	VUS	VUS	VUS	VUS	VUS	LP	VUS(P)	VUS(P)	VUS(P)	VUS(P)	VUS(N)		
**Neurodevelopmental**	**+**	**+**	**+**	**+**	**+**	**+**	**+**	**+**	**+**	**+**	**+**	**9, 100%**	**11, 100%**
Intellectual disability		+					+	+			+	3, 33%	4, 36%
Developmental delay	+	+	+	+	+	+	+	+	+	+	+	9, 100%	11, 100%
Autism spectrum disorder		+								+	+	1, 11%	3, 27%
**Behavioral abnormalities**	**+**	**+**	**+**	**+**	**+**	**+**	**+**				**+**	**7, 78%**	**8, 73%**
Autistic behaviors						+						1, 11%	1, 9%
Acting out					+		+					2, 22%	2, 18%
Aggressiveness							+					1, 11%	1, 9%
Disharmonic behavior	+											1, 11%	1, 9%
Excessive eating				+			+					2, 22%	2, 18%
Hyperactive											+	0, 0%	1, 9%
Oppositional defiant disorder		+	+				+					3, 33%	3, 27%
Reclusive							+					1, 11%	1, 9%
Tantrums				+	+							2, 22%	2, 18%
**Attention disorders**		**+**	**+**		**+**		**+**		**+**			**5, 56%**	**5, 45%**
ADHD		+	+		+				+			4, 44%	4, 36%
Attention issues							+					1, 11%	1, 9%
**Hypotonia**			**+**			**+**		**+**		**+**		**3, 33%**	**4, 36%**
Hypotonia						+		+		+		2, 22%	3, 27%
Hypotonia global neonatal			+									1, 11%	1, 9%
**Brain MRI**	**N/D**	**N/D**	**+**			**+**	N/D	**+**	**N/D**	**N/D**		**3, 60%**	**3, 50%**
Dysplastic corpus callosum	N/D	N/D				+	N/D	+	N/D	N/D		2, 40%	2, 33%
Dysplastic posterior bodies of the ventricles	N/D	N/D					N/D	+	N/D	N/D		1, 20%	1, 17%
Ectopic posterior pituitary	N/D	N/D				+	N/D		N/D	N/D		1, 20%	1, 17%
Hypoplastic anterior pituitary	N/D	N/D				+	N/D		N/D	N/D		1, 20%	1, 17%
Superior vermis dysplasia	N/D	N/D	+				N/D		N/D	N/D		1,20%	1, 17%
White matter signal alterations	N/D	N/D					N/D	+	N/D	N/D		1, 20%	1, 17%
**Strabismus**			**+**			**+**	**+**					**3, 33%**	**3, 27%**

Abbreviations: ADHD = attention deficit hyperactivity disorder, F = female, LOF = loss‐of‐function, M = male, N/A = not applicable, N/D = not done or not reported, y = years, + = reported, ? = unknown.

^a^ Is also heterozygous for a de novo *CRMP1* c.1052T>C, p.F351S [NM_001014809.2] VUS(P) (PS2, PM2) variant.

^b^ Is also heterozygous for a *TUBB* c.266A>C, p.(Asn89Thr) [NM_178014.4] VUS(N) (PM2) variant.

^c^ Is also heterozygous for a maternally inherited 150 kb 16p11.2 duplication (chr16:28869960–29019738, GRch38; VUS 1A, 4M (0.45)) and a paternally inherited 11p14.1 deletion (chr11:28163792–28326390, GRch38; VUS 1A, 3A, 4J, 5C (−0.45)) that affects the *METTL15* gene (pLI = 0).

Other neurologic phenotypes and mood disorders were described, but none were seen in greater than 20% of the cohort (Table S1). Similarly, no individual non‐central nervous system‐associated phenotype was seen in greater than 20% of the cohort with the exception of strabismus (27%, 3/11). No consistent pattern of dysmorphic features was seen.

### 3.1 Subject S1

Subject S1 is a 3‐year, 9‐month‐old male who is heterozygous for a non‐maternally inherited 1.25 Mb deletion (chr5:115221988–116468765; hg38) which affects *PGGT1B* (pLI = 0), *CCDC112* (pLI = 0), *FEM1C* (pLI = 0.42), *TICAM2* (pLI = 0), *TMED7* (pLI = 0.77), *CDO1* (pLI = 0), *ATG12* (pLI = 0), *AP3S1* (pLI = 0.01), *LVRN* (pLI = 0), *ARL14EPL* (pLI = 0), *COMMD10* (pLI = 0), and *SEMA6A* (pLI = 1). A paternal DNA sample was not provided. His family history is notable for two maternal half‐sisters with intellectual disability, one of whom had a normal chromosomal microarray analysis and a normal 485 gene neurodevelopmental deficiency gene panel. No additional information is available regarding his parents or other family members.

He was born at 40 weeks gestation. Over time, he was noted to have developmental delay. He sat at 19 months of age and walked at > 24 months of age. He had speech delay and at 3 years, 9 months of age he could repeat some words. His behavior has been noted to be “disharmonic.” His height, weight, and OFC were within the normal range. His physical exam was notable for postaxial polydactyly of the left hand.

### 3.2 Subject S2

Subject S2 is a 9‐year, 5‐month‐old female of English origin who is heterozygous for a 942 kb deletion (chr5:115696515–116638031) that affects *CDO1* (pLI = 0), *ATG12* (pLI = 0), *AP3S1* (pLI = 0.01), *LVRN* (pLI = 0), *ARL14EPL* (pLI = 0), *COMMD10* (pLI = 0), and *SEMA6A* (pLI = 1). The inheritance pattern of this deletion is unknown. Fragile X and exome sequencing were negative. She was diagnosed with severe speech and language delay, mild global developmental delay, autism spectrum disorder level 2, mild intellectual disability, oppositional defiant disorder, and attention deficit hyperactivity disorder (ADHD). She was noted to have anxiety, emotional dysregulation, and macrocephaly. No other dysmorphic features were noted on examination.

### 3.3 Subject S3

Subject S3 is a 7‐year‐old female who is heterozygous for a 452 kb deletion (chr5: 116216037–116668476; hg38) which affects *COMMD10* (pLI = 0) and *SEMA6A* (pLI = 1). The inheritance pattern of this deletion is unknown. Her parents have intellectual disability. Pregnancy was complicated by gestational diabetes. She was born at term, was large for gestational age, and was noted to have global hypotonia. Over time she was noted to have gross motor and speech delay, ADHD, vestibular challenges, fine motor difficulty, and oppositional behavior. A brain MRI was performed and showed superior cerebellar vermis dysplasia. She has divergent strabismus. At 7 years of age, her growth parameters were normal, and no dysmorphic features were noted on physical exam.

### 3.4 Subject S4

Subject S4 is a 13‐year‐old White female who carries a maternally inherited 296 kb deletion (chr5:116172632–116468765; hg38) that affects *COMMD10* (pLI = 0) and *SEMA6A* (pLI = 1). She was also found to be heterozygous for a de novo *CRMP1* c.1052T>C, p.F351S [NM_001014809.2] VUSP (PS2, PM2) as described by Ravindran et al. [24] This variant may be contributing to her phenotype.

Pregnancy complications included maternal obesity, first trimester bleeding, and maternal hypertension controlled with Trandate (labetalol hydrochloride). She was born at 36 weeks gestation via cesarean section. She weighed 3.601 kg (98th centile, +2.1 SD), was 50 cm in length (92nd centile, +1.4 SD), and had a head circumference of 35.2 cm (98th centile, +2 SD). Apgar scores were 8 and 10 at 1 and 5 min, respectively. Perinatal findings included respiratory distress and stridor. Over time, she was noted to have developmental delay. She sat at 1 year of age, walked at 2 years of age, and spoke her first words at 2.5 years of age. Her current language abilities are poor, and she has temper tantrums. She began to overeat at 2 years of age, and she is currently obese. She had urinary incontinence for several years with no identifiable anatomical cause.

At 13 years of age, she weighed 115 kg (99th centile, +4.9 SD), had a height of 164 cm (83rd centile, +0.9 SD), had a BMI of 42.75 kg/m^2^ (99th centile, +3.3 SD), and had an occipital frontal circumference (OFC) of 59.6 cm (99th centile, +4.7 SD). She was noted to have a square‐shaped face, midface retrusion, an everted “tented” upper lip vermilion, and hyperlordosis.

### 3.5 Subject S5

Subject S5 is a 21‐year‐old African American male who carries a maternally inherited 262 kb deletion (chr5:116226979–116489332; hg38) that affects *COMMD10* (pLI = 0) and *SEMA6A* (pLI = 1). His asymptomatic mother holds advanced degrees and works as an engineer. Pregnancy was complicated by spotting, an undefined infection which was treated with Bactrim, and gestational diabetes controlled with insulin. Prenatal ultrasound examinations were normal. He was born at 38 weeks gestation. He weighed 3.289 kg (62nd centile, +0.3 SD) and spent 5 days in the neonatal intensive care unit (NICU) due to jaundice and bradycardia.

His gross motor development was normal. Specifically, he sat at 6 months, crawled at 7–8 months, and walked at 11 months. His first words were spoken at 10–11 months of age, but he was subsequently noted to have expressive and receptive language delay. He did not speak in sentences until 3 years of age, and he received speech therapy starting at 5 years of age. Over time he was diagnosed with ADHD, bipolar disorder, and depression, and he was noted to have abnormal behaviors including mood swings, tantrums, and acting out.

His most recent physical exam was conducted at 17 years, 1 month of age. His height was 170.2 cm (24th centile, −0.7 SD), he weighed 53.7 kg (10th centile, −1.3 SD), and his OFC was 57.7 cm (93rd centile, +1.5 SD). Features noted on physical exam included a flat affect, a broad, prominent forehead, deep‐set eyes, hypertelorism, a low nasal bridge, and a bulbous nasal tip.

### 3.6 Subject S6

Subject S6 is a 5‐year‐old male of European, Métis, and French‐Canadian heritage. He is heterozygous for a de novo c.231T>G, p.(Tyr77*) variant in *SEMA6A*. Pregnancy was complicated by maternal bleeding. There were no maternal exposures. He was born at term via cesarean section and weighed 3.080 kg (~20th centile, −0.8 SD). Apgar scores were 6 and 9 at 1 and 5 min, respectively. He had neonatal hypoglycemia. He was recognized to have gross motor delay and hypotonia in infancy. He sat between 9 and 12 months and began walking at 35 months of age. His hypotonia gradually improved with time. He could say approximately 100 words between 2.5 and 3 years of age but then lost them and slowly regained them. Currently, he can say short sentences in English and French, can sing the alphabet, and can recognize most letters and some sight words. He does not respond to his name or commands, is difficult to engage, and has autistic behaviors. He has normal growth parameters, delayed tooth eruption, strabismus, doughy skin, and distinctive facial features including a long face, tall prominent forehead, bitemporal narrowing, and thin lips. He was diagnosed with central hypothyroidism and mild ACTH deficiency at 2 years of age and is treated with levothyroxine and glucocorticoid stress dosing for severe illness or surgery. A brain MRI showed a dysplastic corpus callosum, an ectopic posterior pituitary, and a hypoplastic anterior pituitary.

### 3.7 Subject S7

Subject S7 is a 17‐year‐old male who is heterozygous for a maternally inherited c.1254C>G, p.(Tyr418*) stop‐gain variant in *SEMA6A*. He is also heterozygous for a c.266A>C, p.(Asn89Thr) [NM_178014.4] VUS (PM2) in *TUBB* that may be contributing to his phenotype. Heterozygous pathogenic missense variants in *TUBB* are associated with cortical dysplasia, complex, with other brain malformations 6 (MIM# 615771). The inheritance of this variant is unknown.

Pregnancy was uncomplicated with no known exposures, and he was born at 40 weeks' gestation. Apgar scores were 10 and 10 at 1 and 5 min, respectively. At birth, his length was 49.5 cm (20th centile, −0.8 SD), he weighed 3.110 kg (20th centile, −0.8 SD), and his head circumference was 34 cm (68th centile, +0.5 SD). Neonatal findings included cryptorchidism.

Over time, he was noted to have developmental delay and intellectual disability. He was sitting at 15 months, walking at 21 months, and spoke his first words at 15 months. Currently, he has comprehension and pronunciation difficulties, with poorly structured thoughts. He is verbose, jumps from one idea to another, and often revisits the same topics. He is physically and verbally aggressive. He acts out, has anxiety, eats excessively, and has tachyphagia, emotional dysregulation, oppositional defiant disorder, attention issues, and reclusive tendencies. His other medical problems include strabismus and hyperopia. At age 16, he was 1.64 m tall (11th centile, −1.2 SD), weighed 66.7 kg (69th centile, +0.5 SD), had a BMI of 24.8 kg/m^2^ (87th centile, +1.1 SD), and had a head circumference of 54 cm (23rd centile, −0.7 SD). Dysmorphic features noted on exam included microretrognathism, hypertelorism, a prominent nasal bridge, and down slanting palpebral fissures.

### 3.8 Subject S8

Subject S8 is a 14‐year‐old male of Irish, Italian, and White descent who is heterozygous for a paternally inherited c.2098_2101dup, p.(Ser701Lysfs*45) frameshift variant in *SEMA6A*. He is also heterozygous for a maternally inherited 16p11.2 duplication (chr16:28869960–29019738, hg38; VUS 1A, 4M (0.45)) and a paternally inherited 11p14.1 deletion (chr11:28163792–28 326 390, hg38, VUS 1A, 3A, 4J, 5C (−0.45)) that affects the *METTL15* gene (pLI = 0). These CNVs may be contributing to his phenotype. No information is available regarding his parents.

At birth, he weighed 3.090 kg (18th centile, −0.9 SD). Over time, he was diagnosed with developmental delay and intellectual disability. He sat at 18 months, walked at 2 years of age, and could speak about six words at 2 years of age. Currently, his receptive language is more developed than his expressive language. He barely speaks, and when he does, he has a hoarse voice. He is enrolled mostly in special education classes and has an individualized educational plan. His only behavioral issue is a concern for choking. He has nystagmus, alternating esotropia, astigmatism, and wears glasses. He has tonsillar hypertrophy, a submucous cleft palate, mild snoring, and intermittent drooling. Gastrointestinal issues include gastroesophageal reflux disease (GERD). Musculoskeletal issues include scoliosis and hypotonia. Dermatologic problems include alopecia and atopic dermatitis (eczema).

At his most recent physical examination, he was 146.6 cm tall (1st centile, −2.5 SD) and weighed 34.80 kg (< 1st centile, −2.6 SD). He was noted to have started puberty. His neurological exam showed ataxic gait, hypotonia, hyperreflexia, and tight heel cords. His dysmorphic features include residual plagiocephaly, markedly triangular face, light‐colored scalp hair, light‐colored eyebrows, a low set right ear that was posteriorly rotated and cupped, a high nasal bridge, and a thin upper lip. A brain MRI showed extensive abnormality in the white matter of the cerebral hemispheres that was very pronounced on the FLAIR sequence and relatively subtle on the long repetition time (TR)/long echo time (TE) images with no signal abnormality evident on short TR images, suggestive of leukodystrophy. The appearance of the corpus callosum and the posterior bodies of the ventricles suggested possible underlying dysplasia. These findings were stable on repeat brain MRI 4 months later.

### 3.9 Subject S9

Subject S9 is a 16‐year‐old male of German, Western European, and Saudi Arabian heritage who carries a maternally inherited c.1730‐1G>A splice variant in *SEMA6A*. No information is available regarding his parents. Pregnancy was uncomplicated. He was born at 36 weeks gestation and weighed 2.61 kg (6th centile, −1.6 SD). His neonatal course was complicated by respiratory distress and poor suck and swallow requiring a stay in the NICU. Around 3–4 weeks of age, he was diagnosed with dilated cardiomyopathy and has undergone two cardiac transplants. He had failure to thrive, developed oral aversions, and requires gastrostomy tube feeds. He walked at 11 months of age but did not speak until 2.5–3 years. Currently, he communicates well. He has ADHD. Although he has not been formally diagnosed with intellectual disability, he has an individualized education plan (IEP) and requires significant academic support. He has conductive hearing loss in his right ear, a bifid uvula, and a submucous cleft palate. On skin exam, he has moles and café‐au‐lait spots. He has episodes of diarrhea and constipation and has stage III‐IV kidney failure and anemia. His most recent height was 159.60 cm (7th centile, −1.5 SD) and he weighed 37.6 kg (< 1st centile, −2.7 SD).

### 3.10 Subject S10

Subject S10 is a 4.5‐year‐old male who is heterozygous for a de novo c.596G>C, p.(Arg199Pro) single nucleotide variant in *SEMA6A*. Pregnancy was complicated by gestational diabetes and maternal COVID infection, and he was born at 36 weeks gestation. Neonatal findings included hypoglycemia and neonatal respiratory distress syndrome. Over time, he was noted to have developmental delay, autism spectrum disorder, and mixed receptive‐expressive language disorder. His medical history is otherwise unremarkable except for bilateral pressure equalization tube placement. On physical exam he has a broad forehead, thickened helices, mild ptosis, and hypotonia.

### 3.11 Subject S11

Subject S11 is an 11‐year‐old male of Dutch, Turkish, and Surinamese heritage. He carries a de novo c.1996T>G, p.(Ser666Ala) missense variant in *SEMA6A*. His family history is positive for ADHD in his father. There were no complications during pregnancy and no maternal exposures. He was born at term and weighed 3.455 kg (40th centile, −0.25 SD). He has developmental delay, but his gross motor development was normal. Specifically, he sat at 6 months of age and walked at 10 months of age. He was speaking a few words but then lost his ability to speak at 15 months of age. Currently, he makes sounds but has no words. He has severe intellectual disability. He was diagnosed with autism spectrum disorder and was noted to be hyperactive. He has sleeping difficulties for which he takes melatonin. He had a normal brain MRI. His surgical history includes placement of pressure equalization tubes and an adenoidectomy. He follows a gluten‐free diet, has “belly aches” of unknown cause, and constipation. At 10 years, 6 months of age, he was 148.8 cm tall (62nd centile, +0.31 SD) and he weighed 35.5 kg (61st centile, +0.28 SD). He was noted to have a high forehead, a widow's peak, bilateral up‐slanting palpebral fissures, and round ears with simplified helices and a train rail crus.

### 3.12 Protein Modeling

**FIGURE 2:**
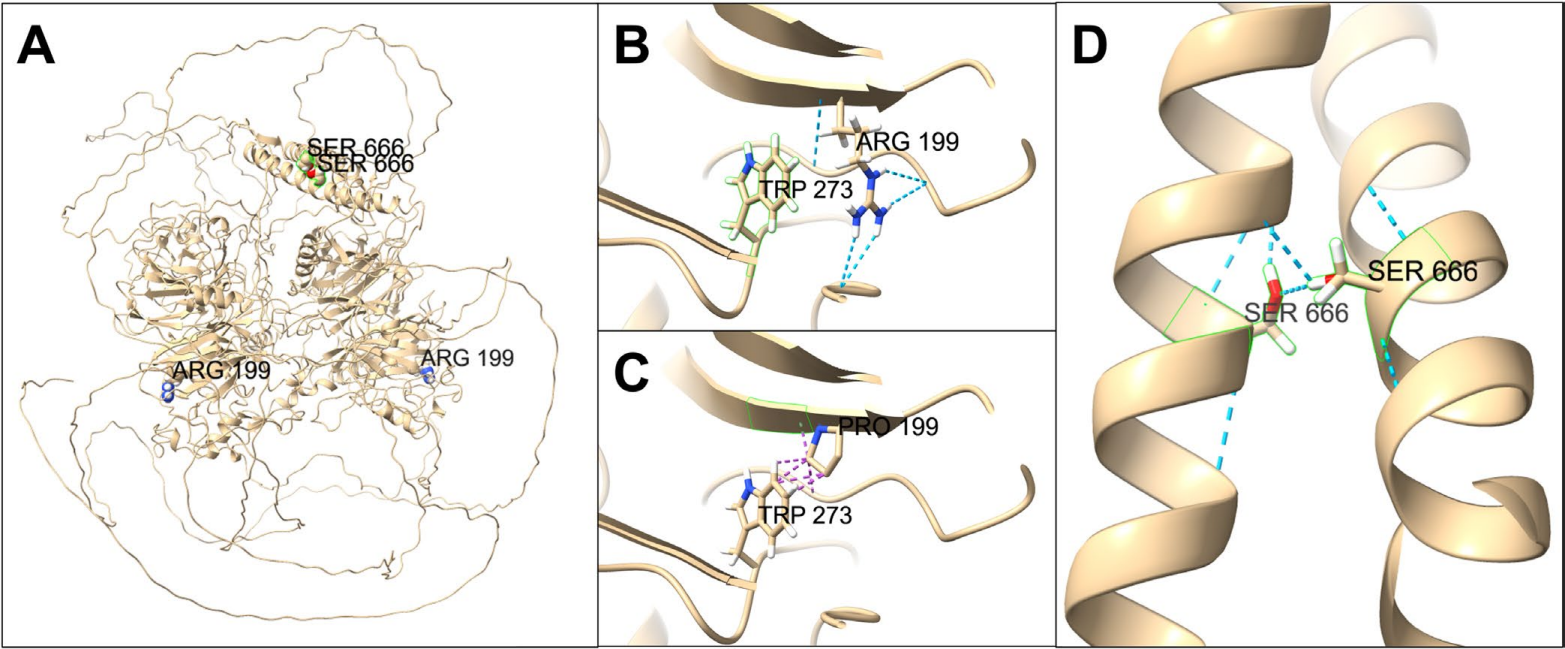
Protein modeling of SEMA6A variants. (A) Modeling of the wild‐type SEMA6A homodimer with the locations of the Arg199 (blue) and Ser666 (green) residues shown. (B, C) Hydrogen bonds between the Arg199 residue and Pro205, Leu207, and Asp261 (blue dashed lines in B) are lost when Arg199 is replaced with a proline, as seen in Subject S10. This replacement results in the gain of six steric clashes with Tyr198, Leu207, and Trp273 (magenta dashed lines in C). (D) The Ser666 residue is located in the transmembrane domain and is predicted to form an alpha helix. The Ser666 residues in the SEMA6A homodimer interact with each and adjacent backbones through hydrogen bonds (blue dashed lines). These hydrogen bonds are lost when Ser666 is replaced by an alanine (amino acid change not shown), as seen in Subject S11. Four intra‐alpha helical hydrogen bonds are also depicted as blue dashed lines.

SEMA6A functions as a homodimer (Figure 2A) [2, 3]. The Arg199 residue is located in the extracellular SEMA domain in a region that is predicted to form a beta pleated sheet (Figures 1B and 2A). Replacement of Arg199 with proline, as seen in Subject S10, results in the loss of five hydrogen bonds—two with Pro205, one with Leu207, and two with Asp261 (Figure 2B). This replacement also leads to six steric clashes—one with Tyr198, one with Leu207, and four with the side chain of Trp273 (Figure 2C).

The Ser666 residue is located in the transmembrane portion of the protein which is predicted to form an alpha helix (Figure 1B, Figure 2A,D). The Ser666 residues of each SEMA6A molecule in the homodimer interact with each other through a hydrogen bond, and their side chains interact with adjacent backbones through two other hydrogen bonds (Figure 2D). The replacement of Ser666 with alanine, as seen in Subject S11, will lead to the loss of those three hydrogen bonds.

## 4 Discussion

### 4.1 Subject S1–S11 Are Heterozygous for Variants That Likely Lead to a Loss of SEMA6A Function

Subjects S1‐S5 are heterozygous for deletions that affect all or a portion of *SEMA6A*. The phenotypes associated with these deletions are most likely to be due to the loss of *SEMA6A* function since it is the only gene affected which is strongly predicted to be haploinsufficient (pHaplo = 0.98, pLI = 1, LOEUF = 0.36) [9, 10, 11].

The phenotypes of Subjects S1–S5 also overlap those documented in Subjects S6–S9 who are heterozygous for loss‐of‐function variants (Tables 1 and S1). It is likely that all of these variants lead to loss of SEMA6A function by triggering nonsense‐mediated mRNA decay (NMD) or producing severely truncated SEMA6A proteins (Figure 1) [25].

It is more difficult to predict the mechanism(s) of action by which the de novo missense variants in Subjects S10 and S11 affect SEMA6A function. However, the p.(Arg199Pro) variant in Subject S10 is predicted to lead to the loss of five hydrogen bonds and to generate six steric clashes (Figure 2B,C). The p.(Ser666Ala) variant in Subject S11 affects the transmembrane domain and leads to the loss of three hydrogen bonds that form between SEMA6A molecules when they form homodimers (Figure 2D). Both variants affect conserved residues, and the phenotypes of Subjects S10 and S11 overlap those of Subjects S1–S9 (Tables 1 and S1). This suggests that these changes may also lead to a loss of SEMA6A function. However, we cannot exclude the possibility that these variants may be acting through a gain‐of‐function or dominant‐negative mechanism.

### 4.2 Neurodevelopmental Phenotypes, Abnormal Behaviors, Disorders of Attention, Hypotonia, and Brain Anomalies Are Recurrently Seen in Subjects S1–S11

Neurodevelopmental phenotypes that include developmental delay, intellectual disability, and/or autism spectrum disorder are seen in 100% (11/11) of Subjects S1–S11 (Tables 1 and S1). Similarly, individual AC05‐0059‐01 reported by De Rubeis et al. had autism spectrum disorder and was heterozygous for a de novo c.27T>A, p.(Tyr9*) stop‐gain variant in *SEMA6A* [12].

Abnormal behaviors were seen in 73% (8/11) of our cohort, with oppositional defiant disorder being diagnosed in 27% (3/11), and acting out, overeating, and tantrums each being described in 18% (2/11). Disorders of attention were documented in 45% (5/11), and hypotonia was seen in 36% (4/11). Among the six individuals who had a brain MRI, 50% (3/6) had at least one abnormal finding, with dysplastic corpus callosum being the only recurrently identified abnormality (33%, 2/6). The only other phenotype seen in greater than 20% of the cohort was strabismus (27%, 3/11), and no consistent pattern of dysmorphic features was documented.

We note that Subjects S4, S7, and S8 carry additional variants that could be contributing to their phenotypes (Table S1). Although these variants are currently classified as VUSs, future studies may warrant a change in their classification to likely pathogenic or pathogenic. In this case, individuals carrying those variants could be considered to have a dual diagnosis similar to female subject DDD4K.03722 from the DDD study who was heterozygous for both a de novo p.(Leu755Profs*74) frameshift variant in *SEMA6A* and a pathogenic variant in *BCL11A*, consistent with a diagnosis of Dias‐Logan syndrome [13, 26].

Subject 9 has several phenotypes not seen in other members of the cohort, including dilated cardiomyopathy and Stage III–IV kidney failure. Although SEMA6A is expressed in most organs based on data from the Human Protein Atlas, the absence of these phenotypes in other individuals in the cohort and in mouse models makes it unlikely that they are caused by a loss of SEMA6A function [4, 5, 6, 7, 8, 27].

### 4.3 Concordance of Human and Mouse Phenotypes

The recurrent phenotypes seen in our cohort are brain‐specific as would be expected based on mouse models. In mice, SEMA6A is required for normal development of the thalamocortical projection and influences proper hippocampal development [4, 5]. *Sema6a*‐null mice are viable and fertile but have widespread cerebral anatomical defects and altered social interactions and working memory [4, 6]. Menzel et al. found that the loss of SEMA6A function in mice led to a loss of GABAergic interneurons in the primary somatosensory cortex, hippocampus, and reticular thalamic nucleus, and hypothesized that such changes could contribute to cognitive, emotional, attentional, sensory, and sleep defects [7]. SEMA6A has also been shown to be critical in proper mouse oligodendrocyte differentiation, offering another explanation as to its diffuse impact on proper brain development and activity [8].

### 4.4 Loss of SEMA6A Function Can Be Seen in Individuals Who Are Mildly Affected or Asymptomatic

The *SEMA6A* variants seen in Subjects S4–S9 were inherited. Although detailed descriptions of the parents of most of these subjects are not available, Subject S5 inherited his deletion from his asymptomatic mother who holds advanced degrees and works as an engineer. This suggests that individuals who are heterozygous for a SEMA6A loss‐of‐function variant may be mildly affected or asymptomatic. Similar patterns of incomplete penetrance and variable expressivity exist for other haploinsufficient genes and various molecular mechanisms have been proposed including variability in the residual function of the remaining allele, common variants affecting other genes, variants in regulatory regions, and epigenetic factors [28, 29, 30, 31, 32].

### 4.5 Future Studies

The identification of additional affected individuals will help to more firmly define the phenotypic spectrum associated with *SEMA6A* haploinsufficiency, may allow the identification of low‐penetrance phenotypes and genotype/phenotype correlations.

Since five of the *SEMA6A* variants reported in our cohort were inherited, and Subjects S4, S7, and S8 carry additional variants that may be contributing to their phenotypes (Tables 1 and S1), population‐based studies may be needed to determine if *SEMA6A* haploinsufficiency is best characterized as an autosomal dominant disorder with incomplete penetrance or as a risk factor for these phenotypes.

Males predominate in our cohort (73%, 8/11) and of the five inherited variants, 80% (4/5) were maternally inherited. If this trend is replicated in future studies, it would suggest that there may be a female protective effect for *SEMA6A* haploinsufficiency similar to that proposed for autism spectrum disorder [33].

## 5 Conclusions

We conclude that loss of SEMA6A function may be associated with an increased risk of neurodevelopmental phenotypes including variable developmental delay, intellectual disability, autism spectrum disorder, abnormal behaviors, disorders of attention, hypotonia, and structural brain anomalies. In keeping with population‐based data, some individuals with *SEMA6A* haploinsufficiency may be mildly affected or asymptomatic.

## Author Contributions

D.A.S. conceived the study. E.B., T.G., and D.A.S. wrote the first draft of the manuscript. X.Z. and N.M.O. were responsible for providing variant interpretations. M.S.A., E.C.K., F.X., X.L., S.R.L., A.P.O., S.B.B., F.P., S.R., B.D., M.K.G., L.H., M.O., K.M.B., K.E.S., M.A.S., H.S., C.J., A.G., H.S., L.F., and D.A.S. accrued subjects and collected clinical and molecular data. J.A.R. is the key holder for the clinical and molecular data identified in the Baylor Genetics clinical database. E.B. and D.A.S. analyzed clinical and molecular data. V.F. performed the protein modeling. All authors reviewed, edited, and approved the final draft.

## Funding

Funding for the DECIPHER project was provided by the Wellcome Trust (grant number WT223718/Z/21/Z).

## Conflicts of Interest

The Department of Molecular and Human Genetics at Baylor College of Medicine derives revenue from clinical laboratory testing conducted at Baylor Genetics. Otherwise, the authors declare no conflicts of interest.

## Supporting information


**Table S1:** Molecular and clinical data.

## Data Availability

The data generated during this study can be found within the published article and Table S1. All variants reported here have been submitted to the ClinVar database (https://www.ncbi.nlm.nih.gov/clinvar).
